# Angiomatous Nasal Polyp Diagnosed by Preoperative Imaging and Successfully Resected by Endonasal Endoscopic Surgery: A Case Report

**DOI:** 10.7759/cureus.18786

**Published:** 2021-10-14

**Authors:** Maho Iemura-Kashiwagi, Masahiro Kikuchi, Sho Koyasu, Yuji Kitada, Akihiko Sugimoto, Hironori Haga, Yuji Nakamoto, Takayuki Nakagawa, Koichi Omori

**Affiliations:** 1 Otolaryngology, Head and Neck Surgery, Uji-Tokushukai Medical Center, Uji, JPN; 2 Otolaryngology, Head and Neck Surgery, Graduate School of Medicine, Kyoto University, Kyoto, JPN; 3 Diagnostic Imaging and Nuclear Medicine, Graduate School of Medicine, Kyoto University, Kyoto, JPN; 4 Diagnostic Pathology, Kyoto University Hospital, Kyoto, JPN

**Keywords:** endoscopic endonasal surgery, fdg pet, mri, paranasal sinus, nose, angiomatous polyp

## Abstract

Angiomatous polyp is a benign, nonneoplastic nasal polyp that accounts for 4-5% of all inflammatory nasal polyps but is rarely reported in the literature. It can grow rapidly and exhibit an aggressive clinical behavior that can simulate malignant sinonasal tumor. We herein report a case of a 13-year-old boy with a rapidly growing angiomatous polyp in the nasal cavity. We had followed up the patient without significant changes for two years, but the tumor had rapidly grown in the last six months. At first, the rapid growth of the tumor and the bone erosion of the maxilla were suggestive of a malignant tumor. However, with preoperative magnetic resonance imaging (MRI) and [18F]-2-fluoro-2-deoxy-D-glucose positron emission tomography imaging findings, we established the corrective diagnosis of an angiomatous polyp. After the diagnostic imaging, we performed an endoscopic endonasal surgery and totally resected the tumor without unnecessary excessive surgery. Recognition of this disease that can mimic malignancy is important to avoid excessive surgery such as en bloc resection by craniofacial approach, and we believe that MRI findings can be helpful for the imaging diagnosis.

## Introduction

Angiomatous polyp is a benign, nonneoplastic nasal polyp that accounts for 4-5% of all inflammatory nasal polyps [1]. It is characterized by extensive vascular proliferation and angioectasia, with regions that are susceptible to vascular compromise, resulting in venous stasis, thrombosis, and infarction [2]. This tumor can simulate other diseases, such as juvenile angiofibroma and even malignant sinonasal tumors, because they present vascular-rich components, sometimes grow rapidly, and cause substantial bone erosion [1,3-5]. However, simple conservative surgical excision is curative, and postoperative recurrence is rare. Therefore, recognition of this disease and establishment of appropriate preoperative diagnosis are important to avoid excessive surgery such as en bloc resection by craniofacial approach [6]. Here, we report a case of angiomatous nasal polyp that grew rapidly and mimicked malignancy, but could be surgically resected by endoscopic endonasal approach under the correct preoperative diagnosis based on imaging findings.

This study was approved by the Ethics Committee of Kyoto University Hospital (R-2805).

## Case presentation

**Figure 1:**
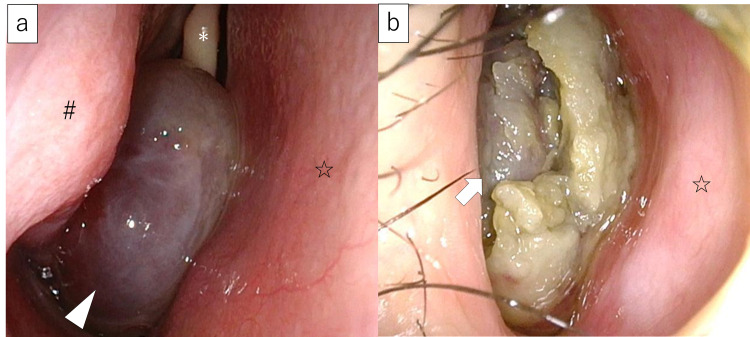
Endoscopic findings in the right nasal cavity (a) Right nasal cavity findings just before the follow-up was discontinued. The tumor was dark reddish in color and localized to the middle nasal meatus (arrow head). (b) Right nasal cavity findings 11 months after the discontinuation of follow-up. The tumor is enlarged and completely occupies the right nasal cavity (arrow). The middle and inferior nasal turbinates are not visible. Necrotic tumor can be observed surrounding the dark red tumor. *Middle turbinate, #Inferior turbinate, ☆Nasal septum.

A 10-year-old male patient without serious medical history was referred to our hospital for an incidentally found nasal tumor on the right side in 2018. The tumor was relatively small and localized to the middle to the common nasal meatus, and the biopsy showed that it was a polyp. Therefore, the patient was followed up in the outpatient clinic for two years, and the size of the tumor did not change. However, the follow-up stopped in January 2020 due to the coronavirus disease 2019 pandemic. In December 2020, he came to the outpatient clinic again because of bilateral nasal obstruction that had worsened over the past six months. No bleeding was observed during the periods the follow-up was interrupted. An endoscopic examination showed that the right nasal tumor was dark reddish in color and enlarged with surrounding necrotic tissue, completely occupying the right nasal cavity and extending into the choana (Figure 1). The tumor was significantly large that it had deviated the nasal septum to the left and deviated the soft palate downward.

**Figure 2:**
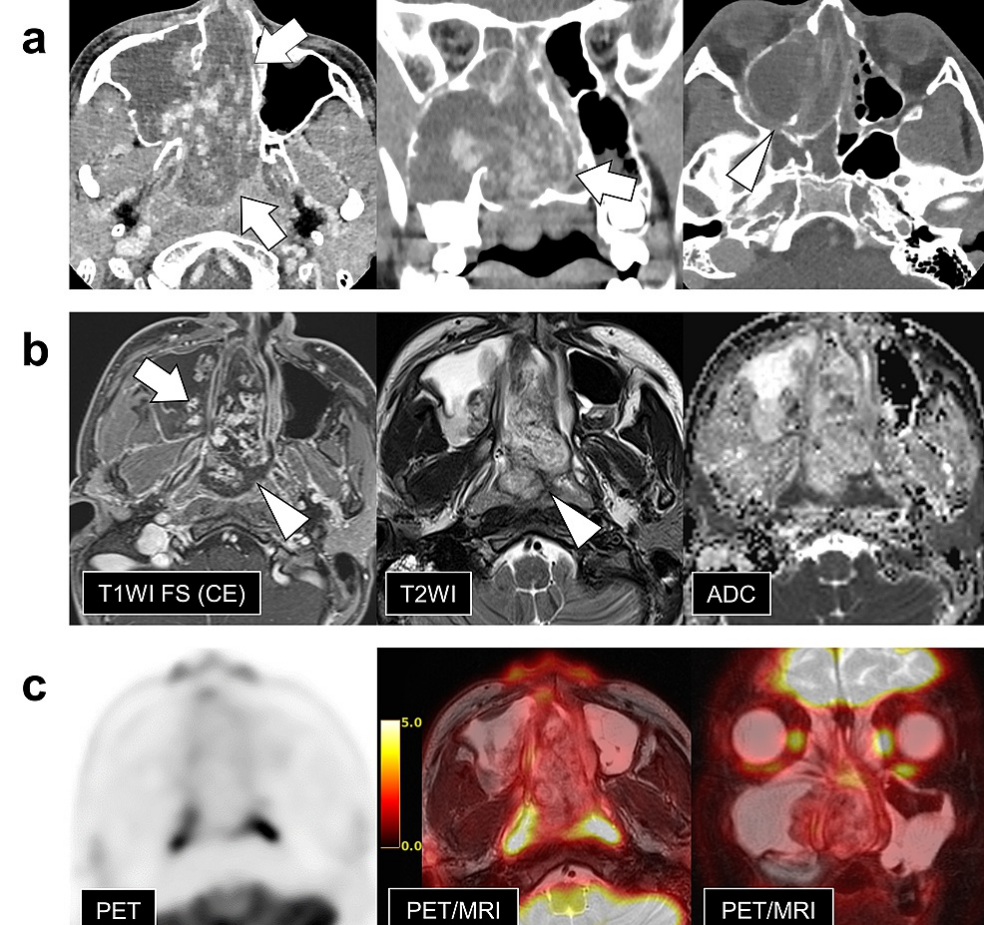
Preoperative imaging findings a) Contrast-enhanced computed tomography (CT), b) contrast-enhanced magnetic resonance imaging (MRI), and c) fused positron emission tomography (PET)/MRI. CT (a) shows a large tumor extending from the right nasal cavity to the nasopharynx (arrows). There is no gross infiltrative bone destruction around the lesion, including the sphenopalatine foramen or pterygopalatine fossa (arrowhead). MRI (b) shows heterogeneously enhanced tumor (white arrow) with unenhanced hypointense rim (arrowheads).The mean apparent diffusion coefficient (ADC) values of the main tumors in the nasal cavity were 2.06×10^-3^ mm^2^/s. PET (c) reveals that the region depicted by T2-weighted imaging shows faint [18F]-2-fluoro-2-deoxy-D-glucose accumulation on PET.

Contrast-enhanced computed tomography (CT) revealed an expansive lesion with a confluent lobular to dendritic-shaped enhancement, occupying from the right maxillary sinus to the right nasal cavity and extending into the right ethmoid sinus and nasopharynx (Figure 2). The lesion dilated the foramen of the maxillary sinus, but there was no gross infiltrative bone destruction around the lesion, including the sphenopalatine foramen or pterygopalatine fossa. On magnetic resonance imaging (MRI), fat-suppressed contrast-enhanced T1-weighted images (CE T1WI FS) showed marked enhancement similar to the CT findings. Although most of the tumor signal on T2WIs was higher than that normally observed in solid tumors, there was peripheral hypointense rim that was unenhanced on CE T1WI. The mean apparent diffusion coefficient (ADC) values of the main tumors in the nasal cavity were rather elevated at 2.06×10^-3^ mm^2^/s. The signal in the maxillary sinus indicated serous fluid collection. Moreover, [18F]-2-fluoro-2-deoxy-D-glucose (FDG)-positron emission tomography/MRI revealed low FDG accumulation throughout the lesion (Figure 2).

Based on imaging findings, we suspected a benign angiomatous polyp, and a surgical resection was planned. Preoperative biopsy was not performed due to the risk of bleeding.

Before surgery, preoperative angiography was performed. Right external carotid angiography showed a slight and partial tumor staining. Sphenopalatine artery was found to be the main feeder; thus, preoperative embolization of both the medial and lateral branches of the artery using coils (Target Nano Helical) and an 8-fold dilution of Embosphere Microspheres (300-500um) was performed.

Three days after the embolization, surgery was performed under general anesthesia in March 2021. The surgery was usual endoscopic endonasal surgery using the one-nostril/one-surgeon/two-handed technique. The tumor was significantly big to perform en bloc resection. Instead, we performed multilayer resection [7].

First, lesions presenting in the nasal cavity and nasopharynx were removed piecemeal. A sample was obtained for frozen section, which confirmed a diagnosis of nonmalignant polyp with immunocyte infiltration. There was no active bleeding during the surgery. Second, right endoscopic modified medial maxillectomy was performed to access the maxillary sinus while preserving the nasolacrimal duct and inferior turbinate [8]. Tumors entered to the maxillary sinus from the natural ostium; however, there was no tumor invasion but only accumulation of the pus in the maxillary sinus. Third, to access the frontal sinus, Draf type IIb procedure was performed. In the frontal sinus, there was only pus accumulation similar to the maxillary sinus. At this stage, we finally found the site that seemed to be the origin of the tumor, the medial wall of the orbit. Subsequently, the remaining tumor was completely resected along with its base. We also resected the lamina papyracea as a safety margin to secure complete tumor resection.

The total blood loss during surgery was 170 ml. We did not insert gauze, but packed absorbable hemostat into the nose. Nasal irrigation with saline solution was started two days after the surgery, and the patient was discharged from the hospital five days after the surgery. The patient’s postoperative course was uneventful, with no bleeding or infection. No symptoms of nasolacrimal duct obstruction were observed.

**Figure 3:**
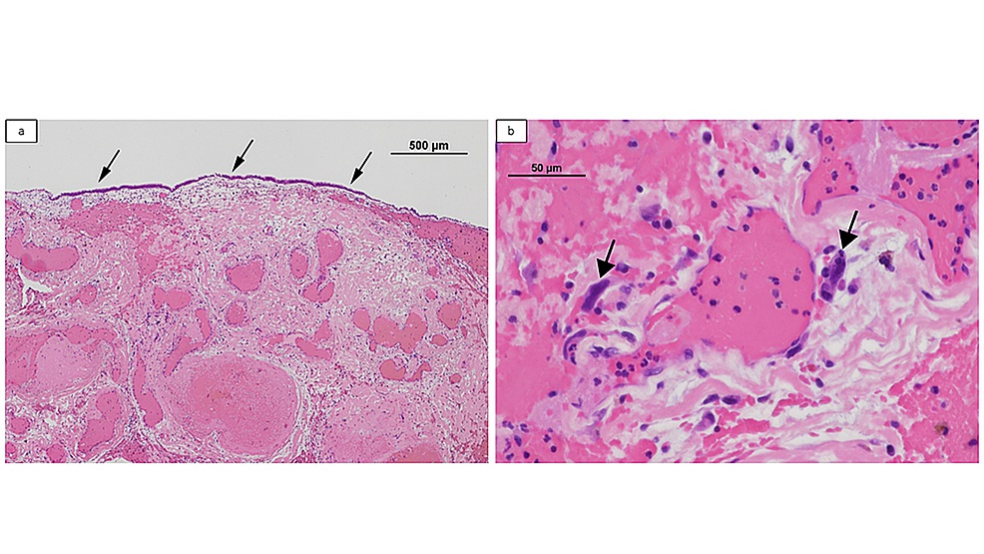
Macroscopic and microscopic findings of surgically resected specimens (a) The lesion is covered with a nonneoplastic respiratory epithelium (arrows). There are abundant dilated blood vessels, hemorrhage, fibrin deposition, and inflammatory cells in the stroma (hematoxylin and eosin [H&E], ×40). (b) Some cells show nuclear enlargement and atypia (arrows), which lack nuclear immunopositivity for androgen receptor and beta-catenin (H&E, ×400).

On histological examination, the tumor was covered with nonneoplastic respiratory epithelium. There were abundant dilated blood vessels, hemorrhage, fibrin deposition, and inflammatory cells in the stroma. Some “stromal” cells showed nuclear enlargement and atypia, which lacked nuclear immunopositivity for androgen receptor and beta-catenin. Accordingly, we finally diagnosed the tumor as an angiomatous polyp. Macroscopic and microscopic findings are shown in Figure 3.

**Figure 4:**
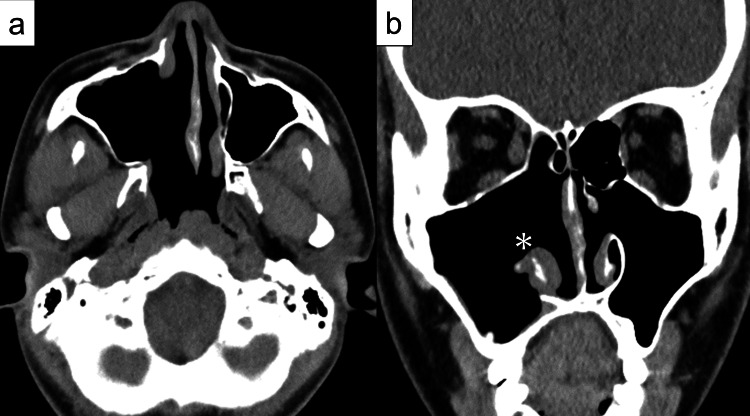
Postoperative computed tomography (CT) findings at 4 months a) Axial image and b) coronal image. CT shows no recurrence of the tumor. *Preserved right inferior turbinate.

Postoperative CT performed four months after the surgery showed no apparent tumor recurrence (Figure 4).

## Discussion

In literature, angiomatous polyp has been most commonly observed in the maxillary sinus with frequent extension into the nasal cavity, choana, and nasopharynx [9,10]. Approximately 90% of angiomatous polyps had origin at the maxillary sinus, and the remaining had the origin at the nasal cavity [3,5,9-12]. The present case had the origin at the medial wall of the orbit.

In the present case, based on these imaging findings, we could establish a correct preoperative diagnosis of the disease without histopathological findings and avoiding excessive surgery, although the tumor had rapidly grown, indicating the possibility of malignancy at first. The low FDG accumulation in the entire tumor reduced the probability of a high-grade carcinoma or sarcoma. Although the patient’s age, sex, and location also suggested the possibility of a juvenile angiofibroma, the anatomical site of origin and direction of extension, morphology of the contrast effect, and MRI findings suggested that this was also unlikely.

In addition, MRI findings were consistent with those of previous reports [10,11,13], such as a peripheral hypointense rim on T2WI or the pattern of heterogeneous enhancement, which is presumably related to hemorrhage with hemosiderin deposition around/in the polyp, may be specific findings of an angiomatous polyp [11,13]. The mean ADC value of an angiomatous polyp is higher ([1.75 +/- 0.30] × 10^−3 ^mm^2^/s) than that of a malignant tumor ([1.18 +/- 0.31] × 10^−3^ mm^2^/s) [13]. We also had hemangioma in our differential diagnosis, especially the capillary type. Based on the signal pattern and morphology on T2WI, we thought angiomatous polyp was the more likely differential diagnosis. We believe that these MRI findings are useful for the imaging diagnosis of angiomatous polyps.

The trigger of rapid tumor growth in this case was uncertain; however, minor trauma might cause damage to the feeder vessels and minor bleeding in the polyp, followed by accelerated reactive and reparative changes with neovascularization [11].

The tumor in this case was similar to a vascular-rich juvenile angiofibroma, which is found in the nasal cavity in adolescent boys and could not be ruled out in appearance. However, the contrast effects on CT and MRI were not significantly high that it was considered unlikely that the tumor was juvenile angiofibroma.

In fact, the angiography before surgery showed only a slight and partial tumor staining.

Dai et al. have reported that 31 patients with angiomatous polyp were treated by surgery alone with no significant blood loss [3]. Chaudhary et al. have reported a case of an angiomatous polyp with blood supply from the internal maxillary artery, which could have been detected with contrast-enhanced CT before surgery, with the absence of active bleeding during surgery [14]. In contrast, Goyal et al. have reported that they experienced a large amount of intraoperative blood loss, which required three units of blood transfusion in the case which showed enhancement in CT imaging [15]. According to these previous reports, angiography and preoperative embolization may not be necessary in all cases, but sometimes well worth considering. In this case, angiography and preoperative embolization may not have been necessary because CT showed only a slight and partial enhancement. Additionally, biopsy could have been performed safely, so even when angiomatous polyp is suspected on imaging, preoperative biopsy should be performed to differentiate between benign and malignant.

## Conclusions

In conclusion, we report a case of large angiomatous polyp that could have been diagnosed with preoperative imaging findings and could be successfully resected by endoscopic endonasal surgery. Since angiomatous polyps are benign tumors usually with little blood supply, it is important not to mistake it for angiofibroma or malignancy, to consider biopsy after evaluation of blood flow, and to avoid excessive surgery such as en bloc resection by craniofacial approach when angiomatous polyps are suspected on imaging and/or histological findings.
